# Drug2Gene: an exhaustive resource to explore effectively the drug-target relation network

**DOI:** 10.1186/1471-2105-15-68

**Published:** 2014-03-11

**Authors:** Helge G Roider, Nadia Pavlova, Ivaylo Kirov, Stoyan Slavov, Todor Slavov, Zlatyo Uzunov, Bertram Weiss

**Affiliations:** 1 Bayer Pharma AG, Müllerstr 178, 13342 Berlin, Germany; 2 Metalife AG, Im Metapark 1, 79297 Winden, Germany

**Keywords:** Drug-target relations, Compound-protein relations, Drug development, Drug discovery, Drug repositioning, Knowledge base, Tool compounds, Biological effect, Bioactivity

## Abstract

**Background:**

Information about drug-target relations is at the heart of drug discovery. There are now dozens of databases providing drug-target interaction data with varying scope, and focus. Therefore, and due to the large chemical space, the overlap of the different data sets is surprisingly small. As searching through these sources manually is cumbersome, time-consuming and error-prone, integrating all the data is highly desirable. Despite a few attempts, integration has been hampered by the diversity of descriptions of compounds, and by the fact that the reported activity values, coming from different data sets, are not always directly comparable due to usage of different metrics or data formats.

**Description:**

We have built Drug2Gene, a knowledge base, which combines the compound/drug-gene/protein information from 19 publicly available databases. A key feature is our rigorous unification and standardization process which makes the data truly comparable on a large scale, allowing for the first time effective data mining in such a large knowledge corpus. As of version 3.2, Drug2Gene contains 4,372,290 unified relations between compounds and their targets most of which include reported bioactivity data. We extend this set with putative (i.e. homology-inferred) relations where sufficient sequence homology between proteins suggests they may bind to similar compounds. Drug2Gene provides powerful search functionalities, very flexible export procedures, and a user-friendly web interface.

**Conclusions:**

Drug2Gene v3.2 has become a mature and comprehensive knowledge base providing unified, standardized drug-target related information gathered from publicly available data sources. It can be used to integrate proprietary data sets with publicly available data sets. Its main goal is to be a ‘one-stop shop’ to identify tool compounds targeting a given gene product or for finding all known targets of a drug. Drug2Gene with its integrated data set of public compound-target relations is freely accessible without restrictions at http://www.drug2gene.com.

## Background

High-throughput screening techniques caused a dramatic increase in drug-target related information not only within pharmaceutical companies but also in public databases. For instance, as of September, 2013, ChEMBL [1] contained 12,077,491 bioactivity evidences, 1,324,941 compounds, and 9,356 protein targets [2]. BindingDB [3] grew from around 20,000 drug-target binding activities in 2007 to 620,000 as of January 2013 [4] and the number of relations in the current version 3 of DrugBank has expanded by more than 50% compared to the previous release [5]. Adequate consolidation and exploration of this compound-gene relation space can have direct impact on the different phases of the drug discovery process by speeding up the identification of tool compounds or by facilitating the repositioning of known drugs [6]. To address this need there exist now numerous databases like STITCH [7], SuperTarget [8], SLAP [9], Dr. PIAS [10], PROMISCUOUS [11], DrugMap Central [12], PiHelper [13], and ChemMapper [14] that offer different levels of representation, curation and annotation of relational data. For example STITCH is an online resource that focuses on interactions between proteins and chemicals. STITCH currently integrates connections between more than 300,000 compounds and 2,600,000 proteins from 1,133 organisms, all extracted from source databases like, ChEMBL, and BindingDB. In addition this data is enriched with protein-protein interaction and biological pathway information.

The availability of such a considerable number of projects built upon the source databases confirms the need of consolidation and improved representation of the compound-target data. However, the scope and functionality of the derived databases is oftentimes specific (single purpose applications) and their data content may not be standardized or normalized. The main motivation to create Drug2Gene is to provide the largest, standardized, and unified compound-target knowledge base that eliminates redundancy to eventually enable effective data mining of the drug-target space.

The Drug2Gene building process integrates data from 19 public bio- and chemo informatics resources, some of which are integrated for the first time in a relation-centered knowledge base. It removes redundancy in the relational, target, and compound namespaces and also facilitates the comparison of experimental data from different sources by standardizing bioactivity data. Drug2Gene enriches the combined dataset with additional homology-based relations through the use of gene homology groups from NCBI HomoloGene [15]. This relational data is paired with powerful search functionality and a user-friendly web interface. Drug2Gene aims at being not only a starting point for, but also a continuous companion during the drug discovery process. Its relation-centered design concept keeps the resource sparse on additional information about interacting partners and instead provides external links to primary sources of compound/drug and gene/protein information.

## Construction and content

### The need for data integration

Due to the ever growing amount of published compound-target interaction data large public compound-target databases that use manual text-mining as their major data source (BindingDB, ChEMBL) increase their coordination efforts by concentrating on different sets of scientific journals [16]. As a result, the overlap between publicly available databases is constantly decreasing. Currently, several source databases have to be searched to comprehensively cover the publicly available drug-target space (see Table 1). In addition, different databases tend to use different naming schemes for genes and compounds and provide measurements of the interaction data in different value formats. As a consequence the search and subsequent unification of the retrieved data requires a considerable amount of time and is highly unpractical. Drug2Gene tackles these problems by providing a single access point to more than 4,000,000 publicly available compound-target interactions. All source datasets are integrated in a universal data structure and presented in a user interface that provides and extends the essential functionalities of the primary databases.

**Table 1:** Drug2Gene data source statistics

**Source database**	**Genes/proteins**	**Drugs/compounds**	**Relations/interactions**	**Unique relations in DB**
CGDCP [17]	6071	3115	169154	154532 (3.534%)
ChEMBL [1]	5115	746582	2830526	2519174 (57.617%)
CTD [18]	27314	11569	89094	70944 (1.623%)
DrugBank [5]	3726	7825	17321	7338 (0.168%)
IUPHAR [19]	114	1455	651	348 (0.008%)
MICAD [20]	249	68	70	55 (0.001%)
PDSP_Ki [21]	605	5256	22790	11505 (0.263%)
PharmGKB [22]	22677	3630	78317	73064 (1.671%)
TTD [23]	1518	2418	2599	1077 (0.025%)
Uniprot [24]	86605	3693	351189	342495 (7.833%)
Ligand Expo [25]	-	7516	32511	26249 (0.600%)
HGNC [26]	24726	-	-	-
PDBsum [27]	23745	-	-	-
ChEBI [28]	-	5015	-	-
NCBI PubChem Compound [29]	-	746546	-	-
NCBI PubChem Substance [29]	-	767740	-	-
PubChem Bioassay [30]	-	-	1124637	831359 (19.014%)
Unified relations shared among two or more DBs	-	-	-	334150 (7.642%)
Total counts from all source databases	202465	2312423	4945372	4372290 (100.00%)
NCBI Gene/Entrez Gene [15]	*Used for Unification/Data Integration*			
Homology inferred relations from NCBI HomoloGene [15]	-	-	226513	-
Total relations in Drug2Gene including homology-inferred relations	-	-	5171885	4598803

Drug2Gene is built of three entities: genes, compounds, and relations between them. The entities are extracted from 19 public source databases. Numbers of entities are listed by source database and by type of the extracted entity - genes, compounds, or relations. Most of the compound-target oriented (relational) databases provide all three types of entries in their flat files. There are also only gene-centered (HGNC, PDBsum, and NCBI Gene) and compound-centered (ChEBI, NCBI PubChem Compound, and NCBI PubChem Substance) source databases that provide only gene or compound entries. Their entities are linked by relational sources like Ligand Expo (links to proteins in PDBsum) and PubChem Bioassay (relations between genes in NCBI Gene and compounds/substances in NCBI PubChem Compound/Substance). Only entries participating in a relation are counted in this table. For data regardless participation in relations, see Additional file 1: Table S2. Dataset - November 2013. The last column shows the number of relations after data integration (and as percent of the total number of integrated relations) unique to each source database.

### Drug2Gene entry: a compound, a gene, and the relation between them

**Figure 1:**
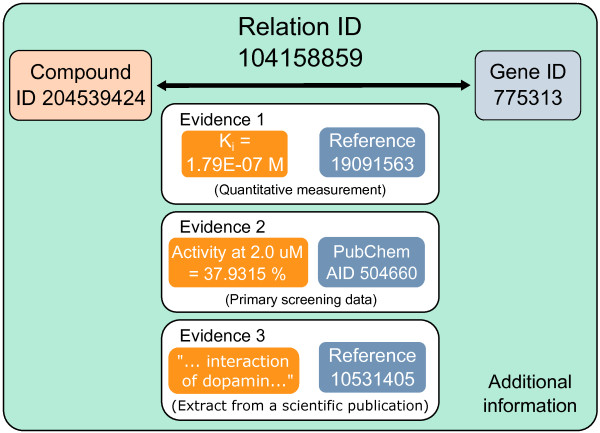
**A Drug2Gene entry.** Conceptually, a Drug2Gene entry consists of a gene, a compound, and a relation between them supported by several evidences. Evidences may come from one or more source databases and therefore be of different types, may have references or assay descriptions. Each entry contains additional information about the unification procedures, links to source database entries, and multiple Drug2Gene annotations.

The concept of Drug2Gene is simple and organized around three main types of entities that together make up a Drug2Gene entry: a compound, a gene, and a relation between them (see Figure 1). Such a relation may be reported by more than one source database and may be supported by more than one evidence. An evidence can thereby be biological activity data, quantitative measurement of binding affinity (i.e. the half maximal inhibitory concentration - IC_50_, the absolute inhibition constant - K_i_, the dissociation constant - K_d_), primary screening data (e.g. activity at given concentration), or other types of qualitative or quantitative data. A sentence or a paragraph from a publication may also be considered an evidence (e.g. “Orlistat is a new inhibitor of pancreatic lipase enzyme.”). Thus, the major function of a Drug2Gene entry is to integrate the relational, compound, and genetic entities from source databases by merging actually identical entries and to aggregate all evidences supporting this relation irrespectively of data formats and in how many different primary databases they had been reported.

### Integration of primary databases into Drug2Gene entries

In its current version (3.2) Drug2Gene has integrated 19 publicly available source databases (Table 1). Our integration process extracts all relations from these databases through separate automated parsing routines tuned for the format of each source database flat file. In total, we extracted 4,945,372 primary database relations between 2,312,423 compound/drug entries and 202,465 target entries (Table 1). These data were analyzed for redundancy and combined with references to the primary sources in the next stage of the integration process - the unification.

The unification process has two major phases – id-mapping of target entries and detection and merging of chemical entries. The id-mapping of all target entries to gene entries is based on a common index, the Entrez Gene Index from NCBI [15]. Entities mapping to the same NCBI gene are thereby merged while relations whose target cannot be unambiguously traced to a verified gene or gene product are removed. Thus only protein and nucleotide target entries are imported, while pseudogenes, metabolic pathways, disease, and other more general types of target entities are removed. As there are many published or tested chemical compounds that lack unified identifiers such as Chemical Abstracts Service Numbers (CAS Number) or International Chemical Identifiers (InChI), compound unification is a more complex process, and depends on the information that is available about the compounds in each source database. If chemical structures are available they are extracted from Structure-Data Files (SDF) [31] and passed through a series of purging rules (i.e. removal of free water molecules and small independent ions, elimination of repeating molecules from polymers, ignoring information about isotope composition, dipolar bonds, and stereoisomerism). Purged structures that are identical are subsequently merged. In contrast, if no chemical structure is available we try to map the provided compound names to the entries created by our SDF integration through cross-references between the source databases. For example, if the compound aspirin from a given database has a cross-reference to the entry acetylsalicylic acid in another database, then aspirin is merged with acetylsalicylic acid. Finally, for compounds without available chemical structure, or cross-reference between source databases, we try to use other available identifiers such as InChIKeys, brand names, compound formulas, Simplified Molecular-Input Line-Entry System (SMILES), or synonymous names for an exhaustive search which is performed in a hierarchical order (see Additional file 1: Table S1). If any of the identifiers matches a value from an already processed compound then the two entries are merged. The compounds that cannot be merged by above steps are integrated as separate compound entries.

As a result of our gene mapping and compound unification, evidences for a relation either collapse to a single Drug2Gene entry if they refer to the same compound and gene, or they create a unique Drug2Gene entry with its respective evidence(s). The merging procedure behind every composite entry is reflected in the unification confidence provided to the user – with ‘high confidence’ assigned to SDF/InChI based, ‘medium confidence’ for cross-reference-based, and ‘low confidence’ for name-based merging. In addition, all relations are subject to a reliability scoring system based on the type of evidence supporting the relation in the source databases. For example, computationally predicted evidences have a score of 4, measured interactions – 9, and highly curated interactions (e.g. those of approved drugs) - 10. If the interaction has more than one evidence, the highest score is attributed to the entire relation.

Integrated entries are stored in Drug2Gene’s relational database schema designed to meet the requirements of the combined dataset as well as to normalize data coming from different source databases (Additional file 2: Figure S1). Drug2Gene is regularly updated every six months.

### Creation of homology-based relations

If a gene in an existing relation has an ortholog or paralog with >80% amino acid sequence identity between the encoded protein sequences (according to NCBI HomoloGene) then a so called “homology-inferred relation” to the same compound(s) is created for the corresponding gene. Through this procedure we extend the scope of imported relations by adding probable drug targets as homology-based relations can suggest overlooked side effects (interactions) for a given compound or extend the available set of active compounds for a given gene/protein (see Case studies). All relations inferred by homology are clearly earmarked with a red asterisk in the user interface of Drug2Gene. In queries they can be intentionally disregarded or specifically selected through two dedicated search indices (see User interface).

### Standardization of evidences and classification of relations

Besides the identification and merging of redundant protein and compound data, another prerequisite for efficient data-mining of compound-gene relations is the direct comparability of binding measurements. To this end the data fields “Activity Type”, “Activity Value”, and “Activity Units” in Drug2Gene are subjected to standardization.

We first normalize the types of activity found in the source databases. For example, “Inhibitory Concentration” and active concentration “AC” are replaced by half maximal inhibitory concentration “IC50” if the evidence refers to inhibition. Once the activity types are standardized we convert the measurements to standard units according to International System of Units (SI). For instance, if the data source reports values in micromoles, then they are converted to the molar equivalents such as 207.2 μM to 0.0002072 M. In searches or for data exports only those standardized values are used. Changed values are explicitly denoted in the interface with a SI icon, which displays the original values upon click.

Activity measurements having constants like half maximal inhibitory concentration (IC_50_) or the binding affinity (K_i_) are not self-explanatory for users without experience in the field of biochemistry, medicinal chemistry, or pharmacology. For a more user-friendly searching over those different activity types, we evaluated and assigned them discrete and simple binding strength categories (“strong”, “medium”, “weak”, or “no binding”) which we call “Activity Strength”. To this end, the binding strength of each evidence is evaluated according to its activity value. While arbitrary, the applied cutoffs reflect best practice values typically used in pharmaceutical industry. For instance, IC_50_ values = < 10E-08 M get classified as “strong”, values between 10E-08 and 10E-06 M – classified as “medium”, and values between 10E-06 M and 10E-05 M as “weak”. IC50 values > 10E-05 M are considered unspecific and are set to “no” activity. Once all evidences of a relation have been categorized, the relation will inherit the “strongest” term assigned to any of the evidences. We refer to this latter categorization as the “Relation Strength”. Now, a user can just filter for all “strong” interactions, or all relations with “Relation Strength” > “weak” thus abstracting from the different types of bioactivity measurements.

### Results of data integration

**Figure 2:**
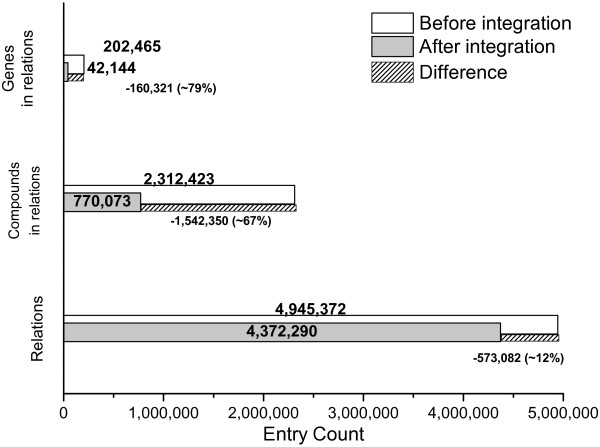
**Integration greatly decreases redundancy compared to searching source databases independently.** Whereas the reduction of redundancy upon integrating data from 19 databases is less prominent for relations (i.e. the different source databases have little overlap (~12%) of relational information), it is very significant especially for the compound space (~67% reduction). Given are number of gene and compound entries and their relations before and after integration together with the difference between these values (the number of entries merged to already existing ones).

Our data integration process unifies target and compound entries. We extracted a total of 202,465 target IDs from source databases which could be mapped by our pipeline to 42,144 unique NCBI gene IDs. This corresponds to a ~79% condensation of the target space. For compounds which participate in relations, unification reduced the number of entries from an initial 2,312,423 entries in the source databases to 770,073 unique entries (~67% reduction) in the Drug2Gene database (see Figure 2). Merging these entities automatically leads to the unification of the corresponding drug-target relations. From the original 4,945,372 source database relations we have assembled 4,372,290 unified relations, corresponding to a ~12% (573,082 entries) decrease (see Figure 2). This decrease is due to an overlap between different relational source databases or from experiments for identical gene-compound interactions within the same source database. From the set of 4,372,290 unified relations produced as a result of the id-mapping and compound unification we predict an additional 226,513 homology-inferred relations using the 43,071 gene homology groups provided by the NCBI HomoloGene databases (Table 1). After integration, we have 4,598,803 unique relations between 42,144 genes and 770,073 compounds that are available in version 3.2 of Drug2Gene.

Genes and compounds not participating in relations are also deposited in Drug2Gene to make sure users always identify a compound or gene in the database even if only to learn there are no relations known so far. Therefore we integrated all gene entries and all compound entries from genetic (e.g. NCBI Gene, HGNC) and chemical (ChEBI, NCBI PubChem) resources (see Additional file 1: Table S2). If these entries are counted, the total number of targets before unification equals 12,168,716 and that of compounds 124,848,656. After integration they are reduced to 11,232,904 (~ 8% reduction) and 28,730,299 (~77% reduction) respectively (see Additional file 3: Figure S2). These normalized compound and gene namespaces are used during updates of the database and for integration of new relational sources.

## Utility and discussion

### User interface

**Figure 3:**
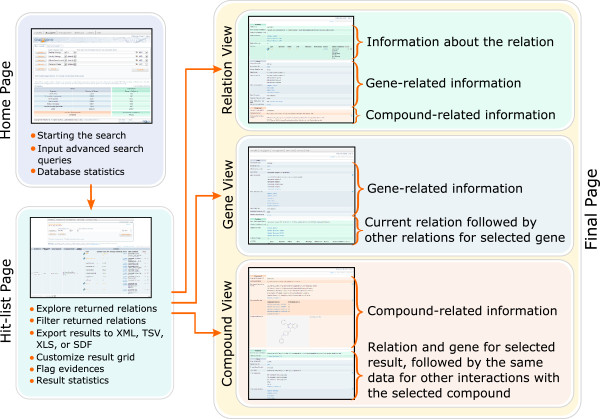
**Drug2Gene search workflow.** The “three-click” search workflow of Drug2Gene consists of “Home page” (enter the search), “Hit-list Page” (result table with option for further filtering, see also Figure 4 for details) and “Final page” (complete information of a single entry). Each page is presented with the basic functionality it provides. The final page comes in three flavors: relation-centric, gene-centric, and compound-centric. In the next version the hit list page will be accompanied by a network visualization. Results can be exported from the hit-list page as well as from the final pages.

Drug2Gene provides an easy three-level web-interface with a “Home page” that holds the search section, a “Hit-list page” for results, and a “Final page” with three entity-oriented views for inspection of specific relations (see Figure 3). A horizontal navigation bar with links to the “Home page” and four other sections (“Management”, “Download”, “Contact”, and “Help”) is displayed on the top of every page. Each search starts from the search section of the “Home page”. This section has a “Main search” and an “Alternative search” mode.

The “Main search” mode permits building complex queries combining up to ten search fields in more than 40 different indices that characterize compound-target relations. Search fields can be combined with Boolean operators (“AND”, “OR”, “AND NOT”). Each index is searched with a specific set of logical operators (“=”, “!=”, “>”, “<”, “>=”, “<=”), defined by its type of data. For text indices the operators “=” (equal) and “!=” (not equal) are available with “strict” (results should match all terms of the search field) or “fulltext” extension (results match any of the terms provided). Some indices are enhanced with an auto-complete function (Additional file 4: Table S3), which reveals their content to the user by suggesting actual values from the indices upon typing. Above the search field the user can also find a help icon which opens an info box showing the type of values that can be entered for each search index. Each field can be expanded to a multiline mode to enter a list of search terms (e.g. all gene IDs of a pathway or a list of compounds of a screen). The use of the main search is illustrated with short examples in the “Case studies” section.

The “Alternative search” has one input field for query strings, either manually written to build more complex and powerful queries or queries saved from previous sessions. The query syntax is simplistic and intuitive, allowing for searches unavailable or too cumbersome to create using the “Simple search” functionality. The current query string created either automatically by using the “Simple search” or explicitly with the “Alternative search” is always displayed at the top of the “Hit list page”.

**Figure 4:**
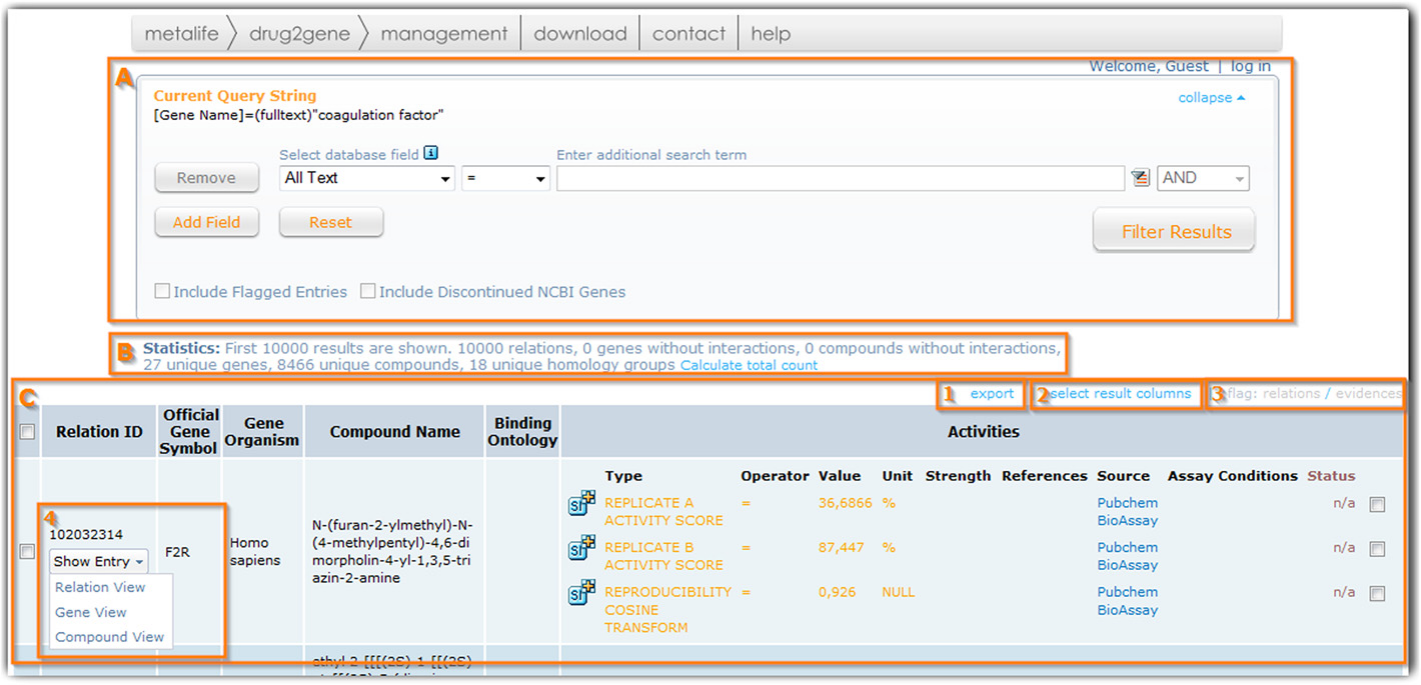
**The “Hit-list page”. A**: Filter section (similar to the query interface with all available indices); **B**: Statistics section (to clarify how many relations have been found between how many unique genes and compounds); **C**: “Results table” with export link (1), table configuration link to add or remove further informative columns (2), and flagging link active only for registered users (3).

The “Hit-list page” provides functionality for effective selection of subsets of target entries via a filter section and a customizable “Results table” (Figure 4A and C). The filter section is functionally identical to the search section on the “Home page”, but applies the new search criteria only on the previous results set. It also displays the currently used query string that can be copied and used in the “Alternative search” mode of the “Home page”. The query syntax is consistent between Drug2Gene versions such that stored queries can be used for documentation purposes or to retrieve an up-to-date result after the database has been updated.

The “Results table” has six columns by default, that present the essential relational information about interacting compounds and genes/proteins together with the available activity information. The number and type of columns in the table is configurable through the “select result columns” link above the upper left corner of the table. Additional columns such as the compound structure can thus easily be added. The first column - “Relation ID”, contains the internal Drug2Gene ID of entries and provides a “Show entry” button (Figure 4, C-4) that leads to the “Final page” which displays, depending on the user choice, either a relation, gene, or compound centric view of the data (Figure 3). A red asterisk (*) next to the ID denotes that the relation has been inferred by homology. If entries are selected with the left-hand check box, they can be exported through the export link in one of the available formats (see “Import and export functionality”). The last column in the “Results table” comprises the evidences ordered into subcategories such as activity values, displayed in base SI units. If the activity data had to be standardized after entering the database, an interactive SI icon is displayed in front of it. Evidences and relations can be flagged as true or false by registered users with the respective rights. Each flag can be supported by comments that are visible for all users of the database. This option is developed with the idea to improve and verify the publicly available relational data through a crowd-sourcing effort.

The “Final page” (Figure 3) displays all available fields for the database entry as well as all the available information for the interacting partners and their relations in one of the three views available through the “Show entry” button.

### Import and export functionality

Import and export of data are indispensable features for a combined knowledge base like Drug2Gene. The import allows the integration of new data sources and their immediate comparison with the existing datasets. At the same time the flexible export guarantees further usability of data beyond the knowledge base.

The data import functionalities of Drug2Gene are situated in the “Management” link on the navigation bar at the top of each page. They are accessible to registered users and allow the import of relational, gene, and compound data in XML, TSV, or SDF format. Sample files are provided for each format. The maximum upload file size is 20 MB and 50 files are allowed per single upload. Batch file import and access to an import API without those restrictions are available by request. Upon import all relations are automatically subject to the unification and standardization procedures of the database. The normalized namespace of compounds and targets (including those without known interactions) is used as a reference during the import of new relations. All these features were developed to handle large datasets. They are constantly used within our IT infrastructure for the import of proprietary datasets.

The export dialog on the “Hit-list Page” (Additional file 3: Figure S3) provides three types of options: export format (XML, TSV, XLS, and SDF in a compressed or uncompressed file), selection of result columns for the export file, and selection of the range of exported results (all results or just those selected by the user). An entry-oriented export is also available in each view of the “Final page” (Figure 3). If the “Compound Structure” checkbox is selected, the molecular structure of compounds participating in the selected relations is included in the export file. The export of compounds to files in SDF format is very useful as the structures can further be used with chemoinformatics programs within various drug development workflows. Structures of individual compounds are also accessible in mol file format through the “download mol file” link in the “Compound Structure” column of the “Results table” and the corresponding category on each view of the “Final page”.

### Case studies

The search section of Drug2Gene provides a user-friendly and flexible query building interface. It empowers scientists of different research backgrounds to effectively search for drug-target related data. The usefulness of Drug2Gene is illustrated by several exemplary workflows described in this section. More detailed step-by-step instructions together with the corresponding query strings are presented in Additional file 5.

### Searching interactions by compound name, formula, or standard identifiers

For example, searching in the compound name index for “zoledronate” returns 59 relations between 55 unique genes and 2 compounds (zoledronate and its salt zoledronate trisodium hydrate). Adding the ‘compound structure’ column to the “Result table” allows visual verification of compound identities. The hit list includes evidences from 7 relational databases – CGDCP, ChEMBL, CTD, DrugBank, PharmGKB, Pubchem BioAssay, and TTD. Their activity types and values are now standardized (“SI” icon in front of the evidence) and directly comparable in the “Results table”. Click on the SI icons to see the original values from the primary resources. If the user prefers only chemically identical matches in the result list (e.g. only the salt), the systematic name or any of the standard identifiers (“InChI”, “InChIKey”, or “SMILES” indices) should be used as a search term. For example a search for the zoledronate salt ZK-thiazolidinone using its InChIKey returns exactly one interaction. InChIKeys are fixed-length character strings fully representing a molecular compound structure. As strings (in contrast to compound structures) can be used in internet search engines they are a very useful start point for chemists or chemoinformaticians to search for a certain compound structure in Drug2Gene or the internet.

In contrast to zoledronate, 1-bromo-2,3-dihydroindole is a compound with no relation data reported, still a user finds the compound in Drug2Gene to distinguish between “compound was not found” and “compound was found but no relations available”.

### Finding all known compounds for a pathway

Researchers often need to quickly understand which compounds are available for a set of genes (e.g. a signaling pathway or the key enzymes of a metabolic pathway).

We start a search for compounds modulating the “Tie2 Signaling” pathway. Pathway Commons provides a list of Gene IDs for this pathway [32], which we enter in the list option searching the ‘Entrez Gene ID’ index of Drug2Gene. The query returns the overwhelming number of 10,646 relations between 6,750 unique compounds and 18 genes. The data is derived from 15,462 evidences coming from 11 primary databases. Most evidences have no bioactivity data (8,684), whereas 6,778 evidences are based on IC50 (3,466), percent inhibition (428), Ki (164), Kd (268), or other activity types (2,452) still relating 4,916 unique compounds to 11 unique genes. Filtering by “Relation Strength” = “Strong” helps to focus conveniently only on the 826 highly active compounds. We may further narrow down the results by filtering for subnanomolar interactions using “Activity Value” < = “1E-09” referring to IC50, EC50, Ki, or Kd (other notations are also accepted, e.g. “0.000000001”, “0.1E-08”, or “10E-10”). This filter returns 93 relations between 86 unique compounds and 6 unique genes. The data (incl. structures and bioactivities) can now be exported as a file in SDF format and loaded into a chemo-informatics workbench to analyze further by chemical properties (e.g. compound similarity clustering, rule of 5, etc.). Another helpful use case showcasing Drug2Gene’s functionality is to explore the target space of a list of compounds, e.g. to evaluate high throughput screening results or to search a whole cluster of structurally similar compounds. We are not aware of any other resource giving this depth of information within a quick query.

### Drug repositioning and predicting side effects

Following the slogan “Drug promiscuity for one can open polypharmacological opportunities for the other”, a very common task in drug repositioning (finding novel applications for already approved drugs) is the exhaustive search for interactions between a given compound and all its intended as well as originally unintended targets (i.e. off-targets). Drug2Gene reduces considerably the time required for gathering such information, which normally is spread between many relational sources.

Thalidomide, a hypnosedative drug, originally prescribed to pregnant women for morning sickness tragically led to fetal malformation of the limbs due to its teratogenicity. The teratogenic effect arises supposedly through inhibition of angiogenesis. However, later Thalidomide was found to be effective against leprosy and in treating multiple myeloma [33]. Searching Drug2Gene for “thalidomide” or its analogs (“pomalidomide”, “lenalidomide”), reports 206 relations and 169 targets including CRBN, the gene conveying thalidomides teratogenic effect. When filtered for “Organism Name” = “Homo sapiens” AND “Relation Strength” > = “Weak” (i.e. at least weak binding to human targets) Drug2Gene selects only 20 relations between four compounds and 14 targets where bioactivity has been convincingly evidenced. Some of these targets are interesting starting points to elucidate the mechanism of thalidomide in e.g. leprosy or multiple myeloma.

Another way in which Drug2Gene can help identify novel avenues for a compound or predict side effects is through its added value of homology-inferred relations. An example illustrating this utility can be found in Additional file 5 (Case study 3). It demonstrates the use of Drug2Gene for the identification of novel inhibitors for metalloproteinase enzymes in human.

### Comparison with other knowledge bases

To our knowledge, the most comprehensive database until now, STITCH, contains over 300,000 compounds in interactions as well as protein interaction data from 1133 species [7]. Other aggregated knowledge bases (e.g. SLAP [9] and PiHelper [13]) offer smaller interaction datasets and restrict their scope to specific subsets of compounds or biological species or optimize their selection of source databases to additional types of data like protein-protein interactions, metabolic pathways, or drug side-effects. In contrast, Drug2Gene aims at providing the most comprehensive set of compound-gene/compound-protein interactions. Currently it contains over 4 million interactions between some 700,000 compounds and more than 40,000 proteins from 1493 organisms (mostly from human, mouse, and rat - see Additional file 3: Figure S4). Importantly, aside from hosting public data, Drug2Gene also allows the upload and integration of proprietary drug-target information. In addition, Drug2Gene provides a unique combination of data content, search functionalities, data export functionalities, and homology based predictions. Its most differentiating feature is the strict unification and standardization of all data allowing for the first time to mine effectively and non-redundantly over the full chemical and biological space including the bioactivity-based evidences. Algorithms and decision rules implemented within the database make use of this standardization by evaluating each bioactivity data to classify the binding strength. With this categorization in place, even inexperienced users without detailed knowledge about IC50, Ki, or Kd may easily differentiate between affine or weak compounds.

Considering the search functionalities, Drug2Gene provides all major types of search options available in other databases - an all text index as well as separate indices for each data category. Instead of several single entity-oriented search interfaces, Drug2Gene has a unique relation-centered interface, which allows the combination of search terms related to different types of entities - genetic, chemical, and experimental. This innovative approach enables the effective harnessing of specific subsets of relations through step-by-step query building. The consecutive filtering of initial search results using binding strength classification or detailed numerical evaluations like IC50 < 10 nM allows the user to assess the effect of each additional criteria and adjust the query according to it. The search supports the full list of Boolean operators (including “AND NOT”) for query construction. Also very complex queries can be built using the “Alternative search” tab or by interrogating Drug2Gene for a full gene and/or compound set using the multiline search mode.

Another distinguishing feature of Drug2Gene is the very flexible export of results available for result sets, for selected entries, or in entry-oriented mode. The export options completely release the data, including compound structures, for use in custom pipelines or special purpose (e.g. chemo-informatics) workflows.

Unlike alternative resources, homology-inferred relations in Drug2Gene are displayed whenever protein similarity suggests a compound may also bind to a hitherto unrelated protein. They can be searched through the indices “Homolog Organism” and “Homolog Similarity” and accessed independently of the drug target relations underlying their creation.

### Future development

Drug2Gene in its current version 3.2 still lacks some features that can be found in other resources, e.g. (sub-) structure-based similarity search many of which we will address with the next release of Drug2Gene. For instance, in the next release 4.0 the effectiveness of searching will be enhanced by allowing to draw and search by chemical structure as well as by structural similarity using Tanimoto distance. Regarding the representation of hit lists we are going to add a graphical network view that allows users to quickly and intuitively navigate through the interaction data, to identify common interaction targets, and to identify possible side-effects.

## Conclusions

We have integrated 19 publicly available source databases and developed a user friendly web-interface that allows not only simple but also highly complex database searches, covering a broad range of user requirements. The current version of Drug2Gene (version 3.2) hosts over 28 million compounds, some 11 million genes, and more than 4 million compound-target interactions. All integrated bioassay data is strictly standardized and normalized, facilitating comparative meta-analyses. This extensive data collection is further extended by homology inferred interactions that can be used e.g. for identifying novel applications for known drugs or predicting unwanted side effects.

On top of this unprecedented data set, we provide extensive features for not only exporting the data in various formats to facilitate subsequent incorporation into third-party scientific research tools but also for uploading user defined datasets.

All of the above mentioned transform the Drug2Gene knowledge base into a ‘one-stop shop’ for identifying tool compounds for genes or pathways or finding all known targets of a drug or to evaluate the biology of the compound hit list of a high-throughput drug screen.

## Availability and requirements

The resource is freely available at http://drug2gene.com. Crowd-sourcing features require registration to enable flagging, annotation of Drug2Gene entries, or upload of own data sets. Authenticated access is provided upon motivated request through the contact section of the site. Due to hardware limitations, a limit of 10,000 entries per single export for guest users exists. Download of larger sets is available upon request (for registered users). All other functionalities are completely accessible without limitation or registration.

## Abbreviations

CAS Number: Chemical Abstracts Service Number; InChI: International Chemical Identifier; SDF: Structure-Data File; SI: International System of Units (abbreviated SI from French, Le Systeme international d’unites); SMILES: Simplified Molecular-Input Line-Entry System.

## Competing interests

HG Roider and B Weiss are employees of Bayer HealthCare AG. N Pavlova, I Kirov, S Slavov, T Slavov, and Z Uzunov are employees of Metalife AG. Drug2Gene is a product of Metalife AG.

## Authors’ contributions

HGR: Conception, database design, software of first prototype (v 0.9), testing, writing of manuscript. NP: coordination, concept development, database design and software of productive Drug2Gene application (v1.0 -3.2), testing, statistics, standardization, writing of manuscript. IK: concept development, testing software of productive Drug2Gene application, case studies, writing of manuscript. SS: development of the server side Drug2Gene application parsers, API. TS: GUI, software of productive Drug2Gene application. ZU: case studies, writing of manuscript. BW: conception, overall design, GUI design, case studies, standardization, testing, writing of manuscript, coordination, funding. All authors read and approved the final manuscript.

## Supplementary Material

Additional file 1: Table S1 Priorities of compound names during compound unification. **Table S2.** Number of gene and compound entries extracted from all source databases regardless their participation in relations. Click here for file

Additional file 2: Figure S1 MS SQL database diagram. Click here for file

Additional file 3: Figure S2 The effect of integration for the entire compound and gene namespaces (regardless their participation in interactions). **Figure S3.** The option menus of the “Results table” on the “Hit-list page”. **Figure S4.** Top-ten most populated species in Drug2Gene by number of relations. Click here for file

Additional file 4: Table S3 Search categories are used to query or filter for a subset of the database. Click here for file

Additional file 5 **Drug2Gene case studies.** Step-by-step instructions. Search steps can be easily reproduced through the advanced query strings provided after each case study. Click here for file

## References

[B1] Gaulton A Bellis LJ Bento AP Chambers J Davies M Hersey A Light Y McGlinchey S Michalovich D Al-Lazikani B Overington JP ChEMBL: a large-scale bioactivity database for drug discovery Nucleic Acids Res 2011 40 D1100 D1107 21948594 10.1093/nar/gkr777 PMC3245175

[B2] ChEMBL 17 release notes ftp://ftp.ebi.ac.uk/pub/databases/chembl/ChEMBLdb/releases/chembl_17/chembl_17_release_notes.txt

[B3] Liu T Lin Y Wen X Jorissen RN Gilson MK BindingDB: a web-accessible database of experimentally determined protein-ligand binding affinities Nucleic Acids Res 2007 35 D198 D201 10.1093/nar/gkl999 17145705 PMC1751547

[B4] BindingDB Website - Info http://www.bindingdb.org/bind/info.jsp

[B5] Knox C Law V Jewison T Liu P Ly S Frolkis A Pon A Banco K Mak C Neveu V Djoumbou Y Eisner R Guo AC Wishart DS DrugBank 3.0: a comprehensive resource for “Omics” research on drugs Nucleic Acids Res 2010 39 D1035 D1041 21059682 10.1093/nar/gkq1126 PMC3013709

[B6] Wassermann AM Bajorath J BindingDB and ChEMBL: online compound databases for drug discovery Expert Opin Drug Discov 2011 6 683 687 10.1517/17460441.2011.579100 22650976

[B7] Kuhn M Szklarczyk D Franceschini A von Mering C Jensen LJ Bork P STITCH 3: zooming in on protein-chemical interactions Nucleic Acids Res 2011 40 D876 D880 22075997 10.1093/nar/gkr1011 PMC3245073

[B8] Hecker N Ahmed J von Eichborn J Dunkel M Macha K Eckert A Gilson MK Bourne PE Preissner R SuperTarget goes quantitative: update on drug-target interactions Nucleic Acids Res 2011 40 D1113 D1117 22067455 10.1093/nar/gkr912 PMC3245174

[B9] Chen B Ding Y Wild DJ Assessing drug target association using semantic linked data PLoS Comput Biol 2012 8 e1002574 10.1371/journal.pcbi.1002574 22859915 PMC3390390

[B10] Sugaya N Kanai S Furuya T Dr. PIAS 2.0: an update of a database of predicted druggable protein-protein interactions Database J Biol Databases Curation 2012 2012 bas034 10.1093/database/bas034 PMC3468816 23060433

[B11] Von Eichborn J Murgueitio MS Dunkel M Koerner S Bourne PE Preissner R PROMISCUOUS: a database for network-based drug-repositioning Nucleic Acids Res 2010 39 D1060 D1066 21071407 10.1093/nar/gkq1037 PMC3013657

[B12] Fu C Jin G Gao J Zhu R Ballesteros-villagrana E Wong STC DrugMap Central: an on-line query and visualization tool to facilitate drug repositioning studies Bioinformatics 2013 29 1834 1836 10.1093/bioinformatics/btt279 23681121 PMC3702253

[B13] Aksoy BA Gao J Dresdner G Wang W Root A Jing X Cerami E Sander C PiHelper: an open source framework for drug-target and antibody-target data Bioinformatics 2013 29 2071 2072 10.1093/bioinformatics/btt345 23766416 PMC3722529

[B14] Gong J Cai C Liu X Ku X Jiang H Gao D Li H ChemMapper: a versatile web server for exploring pharmacology and chemical structure association based on molecular 3D similarity method Bioinformatics 2013 29 1827 1829 10.1093/bioinformatics/btt270 23712658

[B15] NCBI Resource Coordinators Database resources of the National Center for Biotechnology Information Nucleic Acids Res 2012 41 D8 D20 23193264 10.1093/nar/gks1189 PMC3531099

[B16] Nicola G Liu T Gilson MK Public domain databases for medicinal chemistry J Med Chem 2012 55 6987 7002 10.1021/jm300501t 22731701 PMC3427776

[B17] The NCI Cancer Gene Data Curation Pilot https://wiki.nci.nih.gov/display/caBIO/caBIO+Data+Sources#caBIODataSources-CancerGeneIndexProject

[B18] Davis AP Murphy CG Johnson R Lay JM Lennon-Hopkins K Saraceni-Richards C Sciaky D King BL Rosenstein MC Wiegers TC Mattingly CJ The Comparative Toxicogenomics Database: update 2013 Nucleic Acids Res 2012 41 D1104 D1114 23093600 10.1093/nar/gks994 PMC3531134

[B19] Sharman JL Mpamhanga CP Spedding M Germain P Staels B Dacquet C Laudet V Harmar AJ NC-IUPHAR IUPHAR-DB: new receptors and tools for easy searching and visualization of pharmacological data Nucleic Acids Res 2010 39 D534 D538 21087994 10.1093/nar/gkq1062 PMC3013670

[B20] Molecular Imaging and Contrast Agent Database (MICAD) http://www.ncbi.nlm.nih.gov/books/NBK5330/20641331

[B21] Roth BL Lopez E Patel S Kroeze WK The Multiplicity of Serotonin Receptors: Uselessly Diverse Molecules or an Embarrassment of Riches? Neuroscientist 2000 6 252 262 10.1177/107385840000600408

[B22] McDonagh EM Whirl-Carrillo M Garten Y Altman RB Klein TE From pharmacogenomic knowledge acquisition to clinical applications: the PharmGKB as a clinical pharmacogenomic biomarker resource Biomark Med 2011 5 795 806 10.2217/bmm.11.94 22103613 PMC3339046

[B23] Zhu F Shi Z Qin C Tao L Liu X Xu F Zhang L Song Y Liu X Zhang J Han B Zhang P Chen Y Therapeutic target database update 2012: a resource for facilitating target-oriented drug discovery Nucleic Acids Res 2012 40 D1128 D1136 10.1093/nar/gkr797 21948793 PMC3245130

[B24] The UniProt Consortium Reorganizing the protein space at the Universal Protein Resource (UniProt) Nucleic Acids Res 2012 40 D71 D75 22102590 10.1093/nar/gkr981 PMC3245120

[B25] Feng Z Chen L Maddula H Akcan O Oughtred R Berman HM Westbrook J Ligand Depot: a data warehouse for ligands bound to macromolecules Bioinformatics 2004 20 2153 2155 10.1093/bioinformatics/bth214 15059838

[B26] Seal RL Gordon SM Lush MJ Wright MW Bruford EA genenames.org: the HGNC resources in 2011 Nucleic Acids Res 2011 39 D514 D519 10.1093/nar/gkq892 20929869 PMC3013772

[B27] Laskowski RA PDBsum new things Nucleic Acids Res 2009 37 D355 D359 10.1093/nar/gkn860 18996896 PMC2686501

[B28] De Matos P Alcántara R Dekker A Ennis M Hastings J Haug K Spiteri I Turner S Steinbeck C Chemical Entities of Biological Interest: an update Nucleic Acids Res 2010 38 D249 D254 10.1093/nar/gkp886 19854951 PMC2808869

[B29] Bolton EE Wang Y Thiessen PA Bryant SH Chapter 12 PubChem: Integrated Platform of Small Molecules and Biological Activities Annu Reports Comput Chem 2008 4 217 241

[B30] Wang Y Xiao J Suzek TO Zhang J Wang J Zhou Z Han L Karapetyan K Dracheva S Shoemaker BA Bolton E Gindulyte A Bryant SH PubChem’s BioAssay Database Nucleic Acids Res 2012 40 D400 D412 10.1093/nar/gkr1132 22140110 PMC3245056

[B31] Dalby A Nourse JG Hounshell WD Gushurst AKI Grier DL Leland BA Laufer J Description of several chemical structure file formats used by computer programs developed at molecular design limited J Chem Inf Comput Sci 1992 32 244 255 10.1021/ci00007a012

[B32] Cerami EG Gross BE Demir E Rodchenkov I Babur Ö Anwar N Schultz N Bader GD Sander C Pathway Commons, a web resource for biological pathway data Nucleic Acids Res 2011 39 D685 D690 10.1093/nar/gkq1039 21071392 PMC3013659

[B33] Xu M Hou Y Sheng L Peng J Therapeutic effects of thalidomide in hematologic disorders: a review Front Med 2013 7 290 300 10.1007/s11684-013-0277-z 23856973

