# Supplementary material for: Drug2Gene: an exhaustive resource to explore effectively the drug-target relation network
Source: BMC Bioinformatics. 2014 Mar 11;15:68. doi: 10.1186/1471-2105-15-68 (PMC4234465; doi:10.1186/1471-2105-15-68)
Supplement: Additional file 1: Table S1 — Priorities of compound names during compound unification. Table S2. Number of gene and compound entries extracted from all source databases regardless their participation in relations. [file 1471-2105-15-68-S1.pdf]

Table S1: Priorities of compound names during compound unification. During chemical compound unification based on names the following priorities are applied.

1. InChI<br>2. InChI Key<br>3. SMILES<br>4. Formula<br>5. CAS Registry Number<br>6. IUPAC Name<br>7. CAS Name<br>8. Brand Name	9. Generic Name<br>10. Systematic Name<br>11. Traditional Name<br>12. ChEBI Name<br>13. Open Eye Name<br>14. INN<br>15. Mesh<br>16. Synonym

Table S2: Number of gene and compound entries extracted from all source databases regardless their participation in relations. After unification these entries form the namespace used for the creation of new relations (upon proprietary data uploads) or for the update process of existing relations.

Database	Genes/Proteins	Drugs/Compounds
HGNC	781,818	
NCBI Gene	11,232,904	
CGDCP	6,071	2,095
ChEMBL	5115	1,216,322
CTD	27,314	6,690
DrugBank	3,726	6,683
IUPHAR	114	1,796
MICAD	249	116
PDSP_Ki	605	40,154
PharmGKB	22,677	1,777
TTD	1,518	3,190
UniProt	86,605	23,515
ChEBI		20,345
Ligand Expo		10,857
PubChem Compound		35,292,189
PubChem Substance		88,222,926
**Total entries before integration**	12,168,716	124,848,656
**Total entries after integration**	11,232,904	28,730,299
