# Supplementary figures and images for: Drug2Gene: an exhaustive resource to explore effectively the drug-target relation network
Source: BMC Bioinformatics. 2014 Mar 11;15:68. doi: 10.1186/1471-2105-15-68 (PMC4234465; doi:10.1186/1471-2105-15-68)

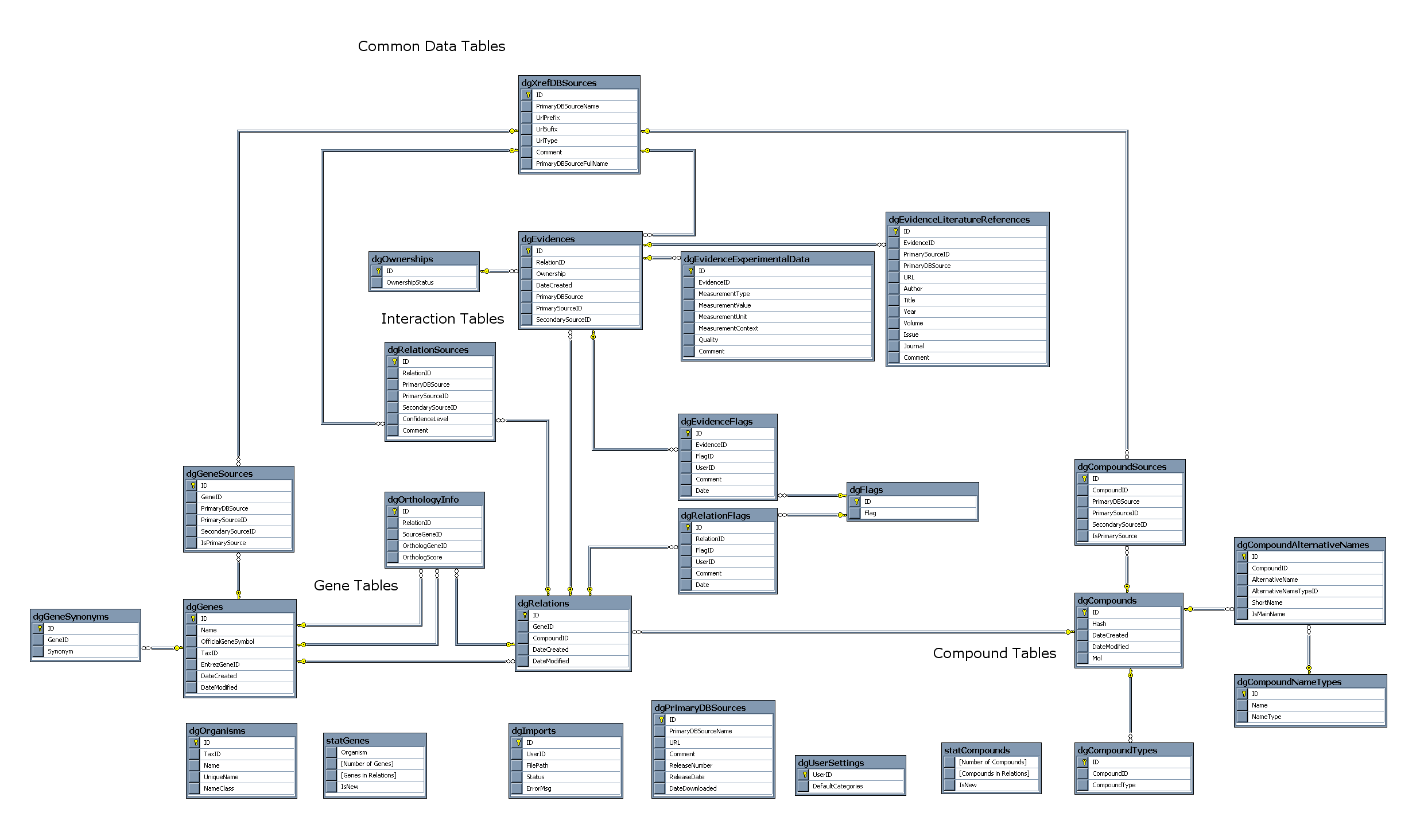

Supplement: Additional file 2: Figure S1 — MS SQL database diagram. [file 1471-2105-15-68-S2.png]
