# Supplementary material for: Drug2Gene: an exhaustive resource to explore effectively the drug-target relation network
Source: BMC Bioinformatics. 2014 Mar 11;15:68. doi: 10.1186/1471-2105-15-68 (PMC4234465; doi:10.1186/1471-2105-15-68)
Supplement: Additional file 3: Figure S2 — The effect of integration for the entire compound and gene namespaces (regardless their participation in interactions). Figure S3. The option menus of the “Results table” on the “Hit-list page”. Figure S4. Top-ten most populated species in Drug2Gene by number of relations. [file 1471-2105-15-68-S3.pdf]

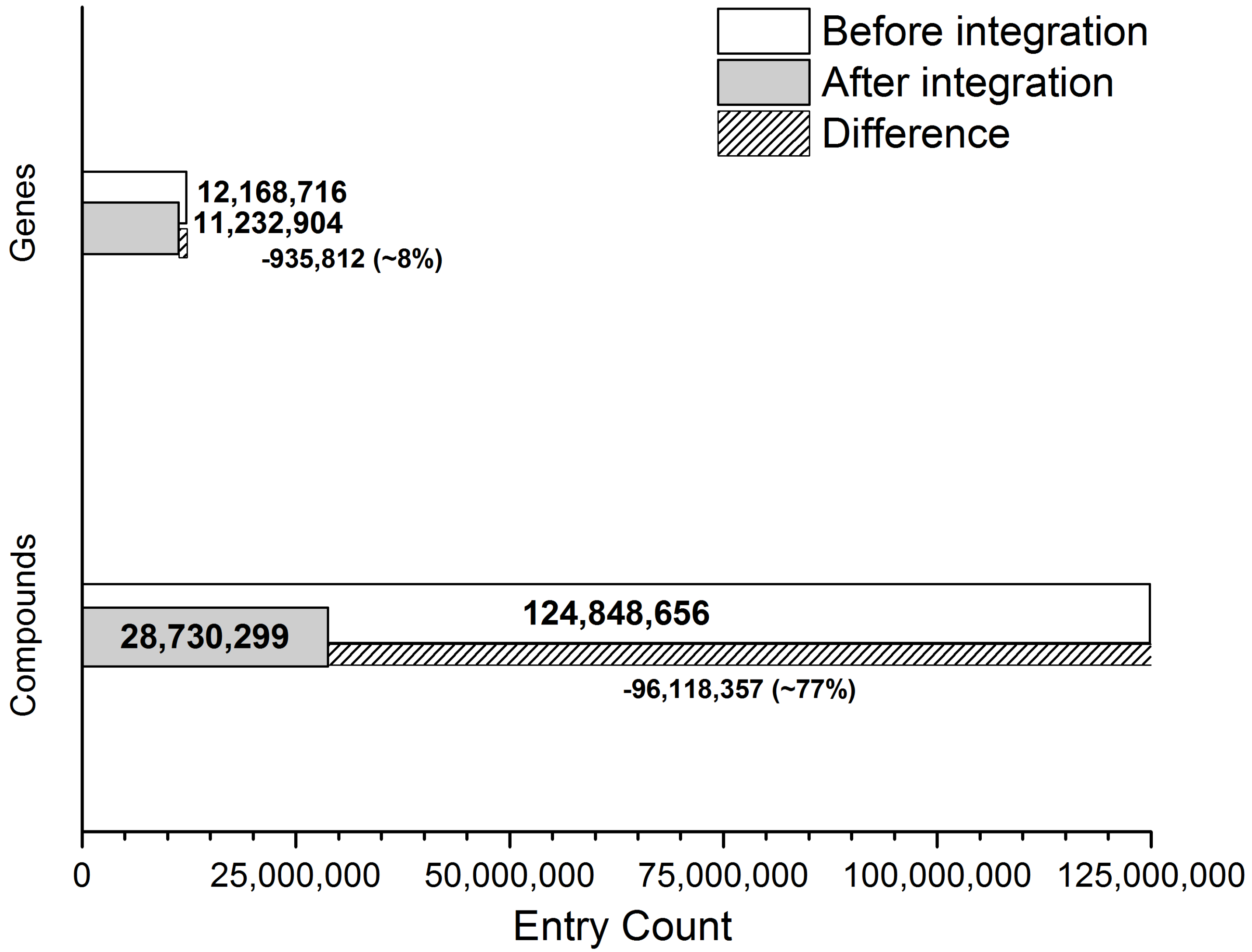

Figure S2: The effect of integration for the entire compound and gene namespaces (regardless their participation in interactions). In Figure 2 the numbers for only those entities that participate in a relation are given.

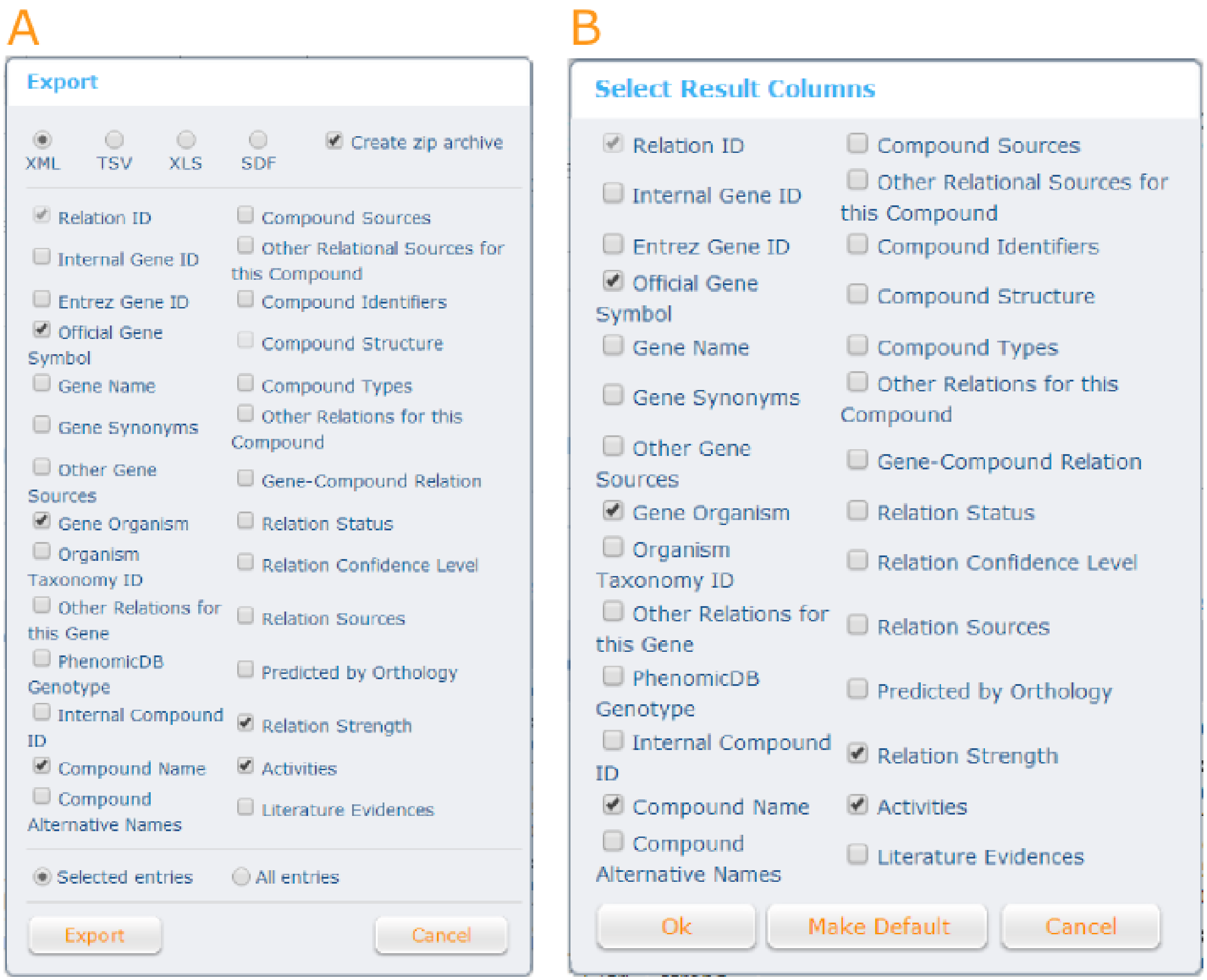

-

Figure S3: The option menus of the "Results table" on the "Hit-list page". **A**: Export options – provide control over which data fields to export and in which format. The user can choose the most appropriate options that fit best the purposes of his further interest; **B**: Select results column – provide control over which data columns to be displayed in the "Results table" of the hit list page.

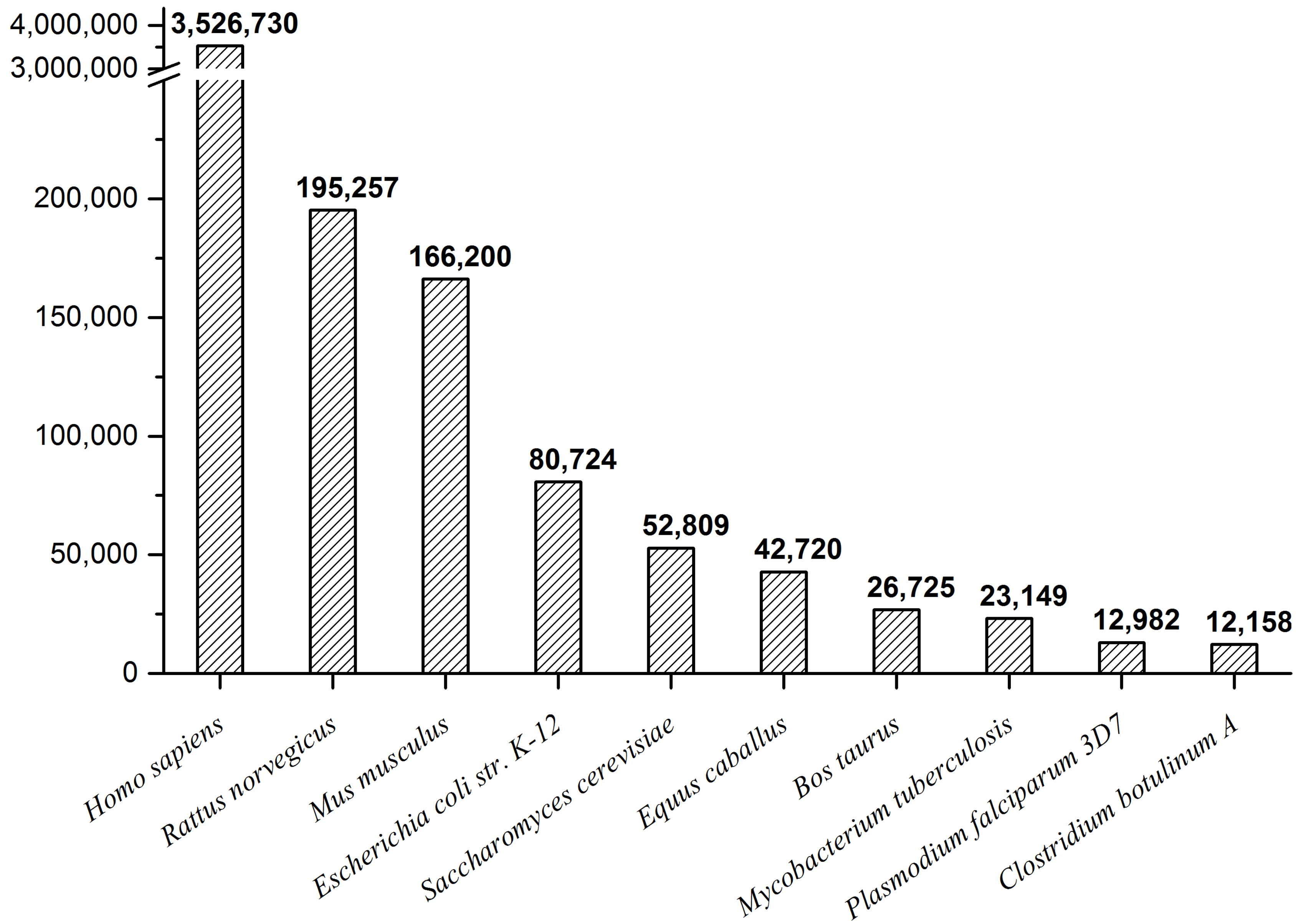

Figure S4: Top-ten most populated species in Drug2Gene by number of relations.
