# Supplementary material for: Drug2Gene: an exhaustive resource to explore effectively the drug-target relation network
Source: BMC Bioinformatics. 2014 Mar 11;15:68. doi: 10.1186/1471-2105-15-68 (PMC4234465; doi:10.1186/1471-2105-15-68)
Supplement: Additional file 4: Table S3 — Search categories are used to query or filter for a subset of the database. [file 1471-2105-15-68-S4.pdf]

Table S3: Search categories are used to query or filter for a subset of the database. A brief description of Drug2Gene's searchable indices is given in the table. **A**: Indices that provide auto-complete functionality; *****: Strict search text indices for which the results must match all terms of the search field.

SEARCH FIELD	DESCRIPTION
All Text	Search in most of the available categories. Useful for searching for terms which cannot be correctly categorized. Too specific or redundant indices are not included (Internal ID, Relation Strength, Flagged evidences, Homolog Organism).
Activity References*	Search in references supporting particular activity evidence (mostly PubMed IDs and DOI IDs). Helps to find e.g. all relations with activities reported in a (set of) publication(s). Very useful to use a PubMed query (e.g. "angiogenesis") to produce a list of PubMed–IDs describing your field of interest and then use Drug2Gene to extract all compounds reported in those publications. Technically this index and the Reference ID index are the same.
Activity Sources*A	Filter for relations coming or not coming from primary sources of the evidences, e.g. ChEMBL, ChEBI, DrugBank. Useful to exclude or restrict to certain primary sources. May give you Drug2Gene functionality e.g. just for ChEMBL.
Activity Status*A	Search in the activities' statuses by value. The available values are: true, false. Activities may have been flagged as true or false by other users (crowd-sourcing annotation). Helps to e.g. exclude relations other users have found to be false (incorrectly captured/reported), or search only for activity values confirmed by other users.
Activity Strength*	Search in the activity strength values, descriptions and types. The available values for the strength are: "strong", "medium", "weak" or "no binding". They represent how strongly a compound has been reported to bind to a target (e.g. IC50 < 10E-8 is strong). Be aware that each evidence reporting some bioactivity data

	is evaluated independently. One relation may have several evidences and hence several (even conflicting) activity strength terms. The “strongest” term is inherited to the relation as “Relation Strength”. That is why the relation strength has always only one strength term and typically is the strength of choice to filter.
Activity Type[*A]	Filter for activity types (e.g. Ki, IC50, and etc.). Typically to be used in combination with “activity unit” and “activity value” to e.g. filter for relations having a IC50=<10E-6 M.
Activity Type (Standard)[*]	Search in activity types converted after the standardization procedures. This index is just a subset of the “Activity Type” index. Here you get rid of many very special activity types (e.g. blood pressure, etc).
Activity Unit[*A]	Search in activity units (nM, M, %, etc.). Keep in mind most data has been normalized and standardized. So stick to SI units whenever possible. But there are also units for blood pressure and other rather unusual activity read-outs.
Activity Value	Search in activity values (integers). Typically, you may enter 10E-9 or similar notation. The ranges depend on the selected activity type.
Assay Conditions	Search in assay conditions. It may contain free text, comments, assay details, etc.
CAS Number	Search in compounds’ CAS (Chemical Abstracts Service) Registry Numbers, e.g. “58-08-2” returns all relations of caffeine.
Chemical Formula	Search in compounds’ chemical formulas, e.g. “C8H10N4O2” for caffeine. Be aware that the identical formulae can still encode different structures.
Compound Name[A]	Search in compounds’ names (all compound synonyms are included too), e.g. search for “punicalin”, or “chromium(2+)” or 1,3,7-trimethylpurine-2,6-dione”

Compound Sources	Search in primary compounds' sources (NCBI PubChem, ChEMBL, and etc). This index allows filtering Drug2Gene for the subset of information coming only from a subset of primary resources. Gives you the possibility to search e.g. ChEMBL content to provide Drug2Gene functionality which ChEMBL does not provide.
Compound Type	Search in compounds' types. Examples of the available values are: Blocker, Drug, Immunosuppressive Agents, and etc. Be aware that for most compounds there is no such information. We have just taken this information from the primary sources where it was available without any standardization. So you cannot use this filter very systematically!
Discontinued Gene IDs*	Used to filter either gene entries with old (discontinued) or entries with current NCBI Gene IDs from the result. The available values are: true, false. Useful when the user has selected to display entries with discontinued Ids (checkbox "Include Discontinued NCBI Genes" option) on the home page, but needs to exclude them (or work only with them) at any of the next steps of query building. Note that entries with deprecated Ids are automatically translated to the new entries when possible (see index "Entrez Gene ID").
Entrez Gene ID	Search in NCBI Gene ids, e.g. '123' for human PLIN2. Translates discontinued NCBI Gene IDs if they have been replaced in later versions of the database. Does not include entries with old (discontinued) Gene IDs in results unless specifically set by the "Include Discontinued NCBI Genes" option.
Flagged Evidences	Includes or excludes relations with flagged evidences from search results. The available values are: true, false. Works also on flags assigned by other users (crowd-sourcing annotation). Has the same effect as the "Include Flagged Entries" option. For searching flags by their value see index "Activity status".
Flagged Relations	Search in relations flagged by the users of Drug2Gene. The available values are: true, false. Relation flags may differ in value from evidence flags. See index 'Flagged

	Evidences' for more information.
Gene Name[A]	Search in genes' names. Use of wild cards may be useful to find e.g. all lysyl oxidases by searching for lysyl oxidase.
Gene Sources	Search in primary genes' sources (HGNC, CTD, and etc). Useful to restrict your search for relations coming from only a subset of primary databases.
Gene Symbols and Synonyms[A]	Search in genes' official gene symbols and gene synonyms. See index 'Gene Synonym' and 'Official Gene Symbol' for more information.
Gene Synonym[A]	Search in genes' synonyms. Contains all non-official gene symbols (may be ambiguous within a species).
Homolog Organism	Search in genes' homologous organism names. Synonyms and common names are also supported. Useful when searching for homology-inferred relations in a specific organism. For example "Homo sapiens" will return all homology-inferred relations in human. These relations can suggest novel compounds with possible activity in human.
Homolog Similarity	Search in genes' homologs percent identical residues. Homology-inferred relations are created only when there is 80% or more amino acid sequence similarity between homologs in different species. Through this index the user can additionally increase the interspecies similarity threshold, e.g. a search for >95 on this index will return only the homology-inferred relations based on more than 95% similarity. This index is also very useful in combination with the "Homolog organism" index.
InChI	Search in compounds' InChI (IUPAC International Chemical Identifier) values, e.g "1S/C8H10N4O2/c1-10-4-9-6-5(10)7(13)12(3)8(14)11(6)2/h4H,1-3H3" returns all relations of caffeine.
Inchi Key	Search in compounds InChIKeys (a fixed-length (27-character) condensed digital representation of an InChI).

	Very useful as an absolute identifier for a compound structure. InChIKeys are often the best identifier in other databases or the web to make sure you search for the identical structure (compound names may be sometimes ambiguous). For example "XXSSHQCOBPQFOL-UHFFFAOYSA-N" is the InChIKey for caffeine.
Internal Compound ID	Search in compounds' unique IDs (internal ids only used within Drug2Gene). Useful for coming back to a compound from an earlier Drug2Gene session. May not be stable, however, over major Drug2Gene releases.
Internal Gene ID	Search in genes' unique IDs (internal for Drug2Gene). Useful for coming back to a gene from an earlier Drug2Gene session. May not be stable, however, over major Drug2Gene releases. Typically, the NCBI gene id is a more reliable gene identifier.
Literature Evidences	Search available publications and articles confirming the particular evidence by bibliographic information – publication title, journal title, year of publication, volume number, issue number etc. Very useful when searching relations reported in a specific journal, or by a specific author or group of authors. For example "Eder C" will return all relations with literature evidences from this author and "European journal of physiology" will restrict the search to this specific journal.
Molecules in Mixture	Contains the number (integer) of how many molecules are in the structure-derived file (sdf) of a compound entry, e.g. salts typically have here a '2' (cation + anion)
Official Gene Symbol[*A]	Search in official gene symbols (these are unique within a given organism. In contrast, non-official gene symbols (often called synonyms) may be ambiguous within a species.
Organism Name[A]	Filter by organism name (being a characteristic of a gene). E.g. filter for 'Homo sapiens' to get only human genes

Organism Taxonomy ID	Search in genes' NCBI Taxonomy IDs. Similar to the index 'Organsim Name', however, here you use the tax id instead of the organism name, e.g. '9606' for 'Homo sapiens'
Reference ID*	Search in the publications IDs (mostly PubMed IDs and DOI IDs). Useful to identify e.g. all relations/compound/gene reported within a certain publication (given they have all been extracted by one of the primary databases). If the user has bibliographic data other than publications IDs (title, year of publication, journal title, authors etc.), the "Literature Evidence" index should be used.
Relation Confidence Level	Search by scoring numbers which refer to the relations' reliability. The score is 'arbitrary' and may be between 0 and 10 (the higher the score the more reliable the relation). This score is assigned to the primary databases by the Drug2Gene Team and shall represent the level of curation (e.g. low databases based on automatic literature extraction, high for manually curated data content. Exception: The Confidence Score of source entries from ChEMBL (between 0 and 9) is adapted to the Relation Confidence Level, i.e. 0 is converted to 1, 1 to 2, and so on.
Relation ID	Search in relations' unique ids. Each relation has a Drug2Gene-internal ID which may not be stable over releases. Useful for quickly coming back to a relation between Drug2Gene sessions.
Relation Sources[A]	Filter by primary relation sources (MICAD, IUPHAR, ChEMBL, etc).
Relation Status	Search in the relations' statuses. The available values are: true, false. If users have annotated all evidences of a relation as being false, then the relation status automatically becomes also 'false'. Useful index to include those relations.
Relation Strength	Search in the relations' strength values. The relation strength term is inherited from the "strongest" activity

	strength term. The available values are: "strong", "medium", "weak" or "no binding". Very useful index filter for relation strength and to abstract from the detailed activity data.
Smiles	Search in compounds' SMILES (Simplified Molecular-Input Line-Entry System), e.g. "CC1=C(C(CCC1)(C)C)CCC(C)CCCC(C)CC(=O)O" for Tretinoin. A SMILES codes for a compound structure similar like an InChI or InChIKey.
