# Supplementary material for: Drug2Gene: an exhaustive resource to explore effectively the drug-target relation network
Source: BMC Bioinformatics. 2014 Mar 11;15:68. doi: 10.1186/1471-2105-15-68 (PMC4234465; doi:10.1186/1471-2105-15-68)
Supplement: Additional file 5 — Drug2Gene case studies. Step-by-step instructions. Search steps can be easily reproduced through the advanced query strings provided after each case study. [file 1471-2105-15-68-S5.docx]

# Drug2Gene case studies: step-by-step instructions

Search steps can be easily reproduced through the advanced query strings provided after each case study.

Case study 1: Searching interactions by compound name, formula, or standard identifiers

1. Start the search by entering “zoledronate” in the search field corresponding to the “Compound Name” index (*Query string 1*). This is one of the four default indices and can be directly used. The query returns 59 relations between 55 unique genes and 2 compounds on the “Hit-list page”. The “Result grid” displays six columns by default. The “Source” subsection of the “Activities” column displays information about the source databases of the evidences – CGDCP, ChEMBL, CTD, DrugBank, PharmGKB, Pubchem BioAssay, and TTD. If available, activity values of evidences are standardized (interactive SI icon is displayed in front of the “Type” subsection).
2. Use the “select result columns” link to display the “Compound Structure” and “Compound Identifier” columns. A click on the header of the “Compound Name” column sorts compounds by name and makes the difference between them more obvious – besides zoledronate there is also a salt of zoledronate - ZK-thiazolidinone with InChIKey: HYMYRPXSMHJPGD-UHFFFAOYSA-A.
3. The InChIKey value can be used to search specifically for chemically identical matches. A filter on the “InChI Key” index with “HYMYRPXSMHJPGD-UHFFFAOYSA-A” returns exactly one interaction (on a relation-oriented “Final Page” view) between ZK-thiazolidinone and polo-like kinase 1 gene (*Query string 2*).
4. Drug2Gene contains also compounds with no relational data. Go back to the “Home page” by selecting the “drug2gene” link on the top navigation bar of the page. A new search for “*1-bromo-2,3-dihydroindole*” in the “Compound Name” index (*Query string 3*) returns exactly one compound without relational data. If a compound is not present in the database, a “No results found!” message is displayed to the user.

Case Study 1 query strings:

Query string 1:

*[Compound Name] =(fulltext) "zoledronate"*

Query string 2:

*([Compound Name] =(fulltext) "zoledronate") AND [InChI Key] =(fulltext) "HYMYRPXSMHJPGD-UHFFFAOYSA-A"*

Query string 3:

*[Compound Name] =(fulltext) "1-bromo-2,3-dihydroindole"*

Case study 2: Finding all known compounds of a pathway

Researchers often need to quickly understand which compounds are available for a set of genes (e.g. a signaling pathway or the key enzymes of a metabolic pathway).

We start a search for compounds modulating the “Tie2 Signaling” pathway.

1. Pathway commons provides a list of Gene Ids by metabolic pathway on [this link](http://www.pathwaycommons.org/pc-snapshot/current-release/gsea/by_species/homo-sapiens-9606-entrez-gene-id.gmt.zip). Download and unzip the file. The extracted file (*homo-sapiens-9606-entrez-gene-id.gmt*) contains tab-separated values. It can be opened with any text editor (e.g. Notepad or Notepad++) or in a spreadsheet program (e.g. Libre Office Calc, or MS Excel). Then use “Find” to go to the line that starts with “Tie2 Signaling”.
2. Copy the list of Gene IDs on that line. If you are using a spreadsheet program you may then paste and transpose the values on a separate sheet, so that each Gene ID is on a separate line/row, and copy them again. In a text editing program you may use “Find and Replace” to substitute the tabulation characters with new lines. Then go on the home page of Drug2Gene.
3. Select the “Entrez Gene ID” index on the first search field, and change the input mode of the field by pressing the “switch to multiple lines” button next to the field.
4. Paste the IDs in the field. As you are working with a relatively small number of values, you may also manually separate them with a new line instead of processing the string in a separate program (see step 2). Start the search with the “Search” button of the search section. The search form will automatically concatenate the values with the “OR” Boolean operator and run the query (*Query string 1*). When the list of values for submission is larger, you may load it from external file and set the appropriate delimiter.
5. The query returns more than 10,000 relations between 6,750 unique compounds and 18 genes. As only the first 10,000 relations are displayed in the “Result grid”, use the “Calculate total count” link in the “Statistics” section, to see the total number of relations in the result (i.e. 10,646). You can explore the result in more detail if you export all entries in xls format through the export link above the “Result grid”. This export format is compatible with popular spreadsheet programs that allow the user to extract additional statistics on the result set. For example in the current result the most evidences have no bioactivity data (8,684), whereas 6,778 evidences are based on IC50 (3,466), Percent Inhibition (428), Ki (164), Kd (268), or other activity types (2,452) still relating 4,916 unique compounds to 11 unique genes.
6. To focus only on highly active compounds, select the “Relation Strength” index and filter by the value “Strong” (*Query string 2*). The hit list contains relations for only 826 highly active compounds (Activity <= 10 nM or active concentration <= 100 nM)
7. To further filter for even subnanomolar compounds, select the “Activity Value” index and search for “1E-09” (other notations are also accepted, e.g. “0.000000001”, “0.1E-08”, or “10E-10”) with the “<=” operator (*Query string 3*). This filter returns 93 relations between 86 unique compounds and 6 unique genes.
8. To export the compounds for further analysis by chemical properties (e.g. compound similarity clustering, rule of 5, etc), use the export link above the “Result grid”. On the option panel the “SDF” radio button should be selected and the “Compound Structure” column checked. Select also the “All entries” radio button. The exported file will include chemical structures.

Case Study 2 query strings:

Query string 1:

*([Entrez Gene ID]="3265" OR [Entrez Gene ID]="51378" OR [Entrez Gene ID]="5781" OR [Entrez Gene ID]="7010" OR [Entrez Gene ID]="6464" OR [Entrez Gene ID]="3845" OR [Entrez Gene ID]="285" OR [Entrez Gene ID]="284" OR [Entrez Gene ID]="5290" OR [Entrez Gene ID]="2888" OR [Entrez Gene ID]="9046" OR [Entrez Gene ID]="5291" OR [Entrez Gene ID]="2886" OR [Entrez Gene ID]="4893" OR [Entrez Gene ID]="2885" OR [Entrez Gene ID]="5295" OR [Entrez Gene ID]="6654" OR [Entrez Gene ID]="5296")*

Query string 2:

*(([Entrez Gene ID]="3265" OR [Entrez Gene ID]="51378" OR [Entrez Gene ID]="5781" OR [Entrez Gene ID]="7010" OR [Entrez Gene ID]="6464" OR [Entrez Gene ID]="3845" OR [Entrez Gene ID]="285" OR [Entrez Gene ID]="284" OR [Entrez Gene ID]="5290" OR [Entrez Gene ID]="2888" OR [Entrez Gene ID]="9046" OR [Entrez Gene ID]="5291" OR [Entrez Gene ID]="2886" OR [Entrez Gene ID]="4893" OR [Entrez Gene ID]="2885" OR [Entrez Gene ID]="5295" OR [Entrez Gene ID]="6654" OR [Entrez Gene ID]="5296")) AND ([Relation Strength]="Strong")*

Query string 3:

*((([Entrez Gene ID]="3265" OR [Entrez Gene ID]="51378" OR [Entrez Gene ID]="5781" OR [Entrez Gene ID]="7010" OR [Entrez Gene ID]="6464" OR [Entrez Gene ID]="3845" OR [Entrez Gene ID]="285" OR [Entrez Gene ID]="284" OR [Entrez Gene ID]="5290" OR [Entrez Gene ID]="2888" OR [Entrez Gene ID]="9046" OR [Entrez Gene ID]="5291" OR [Entrez Gene ID]="2886" OR [Entrez Gene ID]="4893" OR [Entrez Gene ID]="2885" OR [Entrez Gene ID]="5295" OR [Entrez Gene ID]="6654" OR [Entrez Gene ID]="5296")) AND ([Relation Strength]="Strong")) AND ([Activity Value]<="1E-09")*

Case study 3: Drug repositioning: finding novel applications for compounds interacting with a particular type of proteins

A very common task in drug repositioning is the exhaustive search for interactions of a given drug/compound and their target(s) (and their off-targets). What is drug promiscuity (dirty drug) for one is polypharmacology for the other. As outlined in two examples, Drug2Gene reduces considerably the search time as evidence-based drug data is often spread between many relational sources that are now integrated in Drug2Gene.

**Thalidomide**, a hypnosedative drug, used in pregnant women for *morning sickness* led to fetal malformation of the limbs due to its teratogenicity. The teratogenic mechanism is supposedly through inhibition of angiogenesis. Later it was found also useful against leprosy due to being immunostimulatory and only recently against cancer (multiple myeloma).

1. Start the search in Drug2Gene by entering “thalidomide”, “lenalidomide”, and “pomalidomide” as search terms in the “Compound Name” index - either in three separate search fields concatenated with the “OR” Boolean operator (typing is assisted by the auto-complete functionality), or just in one search field in multiple lines mode with each term on a separate line. The query reports 206 relations and 169 targets (*Query string 1*)
2. Add a filter on the “Organism Name” = “Homo sapiens”, and an additional filter field (through the “Add Field” button) on the “Relation Strength” index with operator “>=” and value “Weak”. The query returns all relations to human genes that correspond to at least “Weak” binding – 20 relations between 4 compounds and 14 targets where bioactivity measurement has been convincingly evidenced (*Query string 2*). Interestingly, all targets can be related in downstream literature analyses with angiogenesis, immunostimulation, or cancer: two thyroid hormone receptors, four phosphodiesterase 4 (only weak inhibition), TNF-alpha, two prostaglandin-endoperoxide synthases, aldehyde dehydrogenase, cytochrome P450, methyltransferase, lysine N-methyltransferase, tyrosyl-DNA phosphodiesterase, and Bloom syndrome RecQ helicase-like, a gene associated with recombination-mediated telomere maintenance and breast cancer.

**Metalloproteinase** **enzymes** are involved in pathological processes like arthritis, cardiovascular disease and cancer (Ram et al., Journal of Clinical Immunology, 2006, 26:299–307). As Drug2Gene contains homology inferred relations, they could be used for identification of novel candidate compounds for metalloproteinase inhibitors in human.

1. Start the search by entering “Metalloproteinase” as a value for the “Gene Symbols and Synonyms” index. The result contains 24,574 relations between 5,990 compounds and 16 metalloproteinase targets (*Query string 3*). These results represent the publicly available knowledge from all integrated relational databases. The “Gene Symbols and Synonyms” index is a combination of the columns “Official Gene Symbol” and “Gene Synonym” and returns the broadest possible match of metalloproteinase genes.
2. Select the “Homolog organism” index and write “human” in the available filter field. The filter returns only relations marked with red asterisks (*Query string 4*). These homology inferred relations are one of the added values of Drug2Gene. They transfer knowledge between highly conserved homologs whenever protein similarity is above 80%. The result contains 81 relations between 81 compounds and 7 genes.
3. Use the “select result columns” link to display the “Predicted by Orthology” column. This column displays a link to the homologous gene, the protein similarity on which each relation has been based, as well as the organism of the orthologous gene. These relations come mostly from experiments carried out in rat, mouse, or cattle. The first link leads to a gene-oriented “Final Page” view with all relations for the orthologous gene.
4. The list of 81 candidate inhibitors can be further filtered by activity values. As homology-inferred relations do not directly display activity values of their “model” relations, at this step an export should be used for the generation of a new query that accesses the activity values of “model” relations. Click the “export” link, select the “XLS” radio button, check only the “Official Gene Symbol” and “Internal Compound ID” columns (uncheck the rest of the columns), and select the “All entries” radio button on the bottom of the export options panel. As the export should be relatively small you can also uncheck the checkbox “Create zip archive”. Download the resulting file and open it with your preferred spreadsheet program.
5. In the file you will find three columns – “Relation ID”, “Official Gene Symbol”, and “Internal Compound ID”. Go on the home page of Drug2Gene, select the “Official Gene Symbol” index, and switch the input field to multiline mode. Then copy the gene symbols from that column and paste them in the multiline text field. Repeat the same with the last table column in another search field on the “Internal Compound ID” index. Set the third search field to the “Organism Name” index, type “human” as a search term, set the logical operator “!=(fulltext)” (different than), and start the search. The search form automatically cleans all repeating values from the multiline fields and builds the query string (*Query string 5*).
6. The search returns 88 relations most of which have been used as “model” for the 81 homologous relations in step 4. These relations have activity evidences and now their standardized activity values can be used to reduce the list of 81 candidate inhibitors to compounds with reasonable (strongly evidenced) effect. Set the filter to the “Relation Strength” index and type “Strong” in the text field. The filter results in 61 relations of 56 compounds (*Query string 6*).
7. Display the “Internal Compound ID” column through the “select result columns” link. Now a click on the column header sorts the “Result grid” by Compound ID values and allows to easily identify compounds with broader specificity towards metalloproteinases or such that are highly specific for a particular type of this enzyme. For example compound with ID 200204250 is a strong inhibitor only for mouse Mmp11, but compound with ID 202973554, when used in nanomolar concentrations, binds strongly to MMP13 in cattle and to Adam17 in rat.

Further wet lab analyses can be carried out now to sieve the most appropriate candidates for development of human metalloproteinase inhibitors. Probably most of these compounds have not been further explored in human in the context of their original research and offer novel opportunities within the new context.

Case Study 3 query strings:

**Thalidomide**

Query string 1:

*([Compound Name] =(fulltext) "thalidomide" OR [Compound Name] =(fulltext) "lenalidomide" OR [Compound Name] =(fulltext) "pomalidomide")*

Query string 2:

*(([Compound Name] =(fulltext) "thalidomide" OR [Compound Name] =(fulltext) "lenalidomide" OR [Compound Name] =(fulltext) "pomalidomide")) AND ([Organism Name] =(fulltext) "Homo sapiens" AND [Relation Strength] >= "Weak")*

**Metalloproteinases**

Query string 3:

[Gene Symbols and Synonyms] =(fulltext) "Metalloproteinase"

Query string 4:

([Gene Symbols and Synonyms] =(fulltext) "Metalloproteinase") AND ([Homolog Organism] =(fulltext) "human")

Query string 5:

([Official Gene Symbol]=(strict)"MMP2" OR [Official Gene Symbol]=(strict)"ADAMTS4" OR [Official Gene Symbol]=(strict)"MMP11" OR [Official Gene Symbol]=(strict)"MMP13" OR [Official Gene Symbol]=(strict)"MMP9" OR [Official Gene Symbol]=(strict)"ADAM17" OR [Official Gene Symbol]=(strict)"ZMPSTE24") AND ([Internal Compound ID]="220446096" OR [Internal Compound ID]="207693181" OR [Internal Compound ID]="204211006" OR [Internal Compound ID]="210502462" OR [Internal Compound ID]="219924253" OR [Internal Compound ID]="216767192" OR [Internal Compound ID]="205803208" OR [Internal Compound ID]="204870550" OR [Internal Compound ID]="212828555" OR [Internal Compound ID]="200808052" OR [Internal Compound ID]="206455722" OR [Internal Compound ID]="203197112" OR [Internal Compound ID]="214424783" OR [Internal Compound ID]="221326144" OR [Internal Compound ID]="203880669" OR [Internal Compound ID]="207859716" OR [Internal Compound ID]="201124208" OR [Internal Compound ID]="202973554" OR [Internal Compound ID]="210363393" OR [Internal Compound ID]="224415786" OR [Internal Compound ID]="221090966" OR [Internal Compound ID]="223325108" OR [Internal Compound ID]="206055161" OR [Internal Compound ID]="201201020" OR [Internal Compound ID]="203238155" OR [Internal Compound ID]="201761117" OR [Internal Compound ID]="221642197" OR [Internal Compound ID]="215621362" OR [Internal Compound ID]="214080032" OR [Internal Compound ID]="209206031" OR [Internal Compound ID]="219738948" OR [Internal Compound ID]="211992630" OR [Internal Compound ID]="225643097" OR [Internal Compound ID]="214901316" OR [Internal Compound ID]="205856296" OR [Internal Compound ID]="200603870" OR [Internal Compound ID]="225188075" OR [Internal Compound ID]="202007843" OR [Internal Compound ID]="218926563" OR [Internal Compound ID]="205981400" OR [Internal Compound ID]="205010191" OR [Internal Compound ID]="220179660" OR [Internal Compound ID]="225573776" OR [Internal Compound ID]="204622456" OR [Internal Compound ID]="212595910" OR [Internal Compound ID]="223488691" OR [Internal Compound ID]="223206551" OR [Internal Compound ID]="200386948" OR [Internal Compound ID]="211611806" OR [Internal Compound ID]="210347170" OR [Internal Compound ID]="219003332" OR [Internal Compound ID]="216024094" OR [Internal Compound ID]="201589625" OR [Internal Compound ID]="208925926" OR [Internal Compound ID]="224630859" OR [Internal Compound ID]="206181604" OR [Internal Compound ID]="218639661" OR [Internal Compound ID]="204103693" OR [Internal Compound ID]="209155310" OR [Internal Compound ID]="204723141" OR [Internal Compound ID]="224984930" OR [Internal Compound ID]="209028198" OR [Internal Compound ID]="224795251" OR [Internal Compound ID]="220783915" OR [Internal Compound ID]="204406431" OR [Internal Compound ID]="221699483" OR [Internal Compound ID]="206356743" OR [Internal Compound ID]="200204250" OR [Internal Compound ID]="209268568" OR [Internal Compound ID]="220391617" OR [Internal Compound ID]="213233605" OR [Internal Compound ID]="225108272" OR [Internal Compound ID]="221843159" OR [Internal Compound ID]="204616771" OR [Internal Compound ID]="208280728" OR [Internal Compound ID]="211740751" OR [Internal Compound ID]="210953706" OR [Internal Compound ID]="208672464" OR [Internal Compound ID]="208427772" OR [Internal Compound ID]="205338663" OR [Internal Compound ID]="223082559") AND [Organism Name] !=(fulltext) "human"

Query string 6:

(([Official Gene Symbol]=(strict)"MMP2" OR [Official Gene Symbol]=(strict)"ADAMTS4" OR [Official Gene Symbol]=(strict)"MMP11" OR [Official Gene Symbol]=(strict)"MMP13" OR [Official Gene Symbol]=(strict)"MMP9" OR [Official Gene Symbol]=(strict)"ADAM17" OR [Official Gene Symbol]=(strict)"ZMPSTE24") AND ([Internal Compound ID]="220446096" OR [Internal Compound ID]="207693181" OR [Internal Compound ID]="204211006" OR [Internal Compound ID]="210502462" OR [Internal Compound ID]="219924253" OR [Internal Compound ID]="216767192" OR [Internal Compound ID]="205803208" OR [Internal Compound ID]="204870550" OR [Internal Compound ID]="212828555" OR [Internal Compound ID]="200808052" OR [Internal Compound ID]="206455722" OR [Internal Compound ID]="203197112" OR [Internal Compound ID]="214424783" OR [Internal Compound ID]="221326144" OR [Internal Compound ID]="203880669" OR [Internal Compound ID]="207859716" OR [Internal Compound ID]="201124208" OR [Internal Compound ID]="202973554" OR [Internal Compound ID]="210363393" OR [Internal Compound ID]="224415786" OR [Internal Compound ID]="221090966" OR [Internal Compound ID]="223325108" OR [Internal Compound ID]="206055161" OR [Internal Compound ID]="201201020" OR [Internal Compound ID]="203238155" OR [Internal Compound ID]="201761117" OR [Internal Compound ID]="221642197" OR [Internal Compound ID]="215621362" OR [Internal Compound ID]="214080032" OR [Internal Compound ID]="209206031" OR [Internal Compound ID]="219738948" OR [Internal Compound ID]="211992630" OR [Internal Compound ID]="225643097" OR [Internal Compound ID]="214901316" OR [Internal Compound ID]="205856296" OR [Internal Compound ID]="200603870" OR [Internal Compound ID]="225188075" OR [Internal Compound ID]="202007843" OR [Internal Compound ID]="218926563" OR [Internal Compound ID]="205981400" OR [Internal Compound ID]="205010191" OR [Internal Compound ID]="220179660" OR [Internal Compound ID]="225573776" OR [Internal Compound ID]="204622456" OR [Internal Compound ID]="212595910" OR [Internal Compound ID]="223488691" OR [Internal Compound ID]="223206551" OR [Internal Compound ID]="200386948" OR [Internal Compound ID]="211611806" OR [Internal Compound ID]="210347170" OR [Internal Compound ID]="219003332" OR [Internal Compound ID]="216024094" OR [Internal Compound ID]="201589625" OR [Internal Compound ID]="208925926" OR [Internal Compound ID]="224630859" OR [Internal Compound ID]="206181604" OR [Internal Compound ID]="218639661" OR [Internal Compound ID]="204103693" OR [Internal Compound ID]="209155310" OR [Internal Compound ID]="204723141" OR [Internal Compound ID]="224984930" OR [Internal Compound ID]="209028198" OR [Internal Compound ID]="224795251" OR [Internal Compound ID]="220783915" OR [Internal Compound ID]="204406431" OR [Internal Compound ID]="221699483" OR [Internal Compound ID]="206356743" OR [Internal Compound ID]="200204250" OR [Internal Compound ID]="209268568" OR [Internal Compound ID]="220391617" OR [Internal Compound ID]="213233605" OR [Internal Compound ID]="225108272" OR [Internal Compound ID]="221843159" OR [Internal Compound ID]="204616771" OR [Internal Compound ID]="208280728" OR [Internal Compound ID]="211740751" OR [Internal Compound ID]="210953706" OR [Internal Compound ID]="208672464" OR [Internal Compound ID]="208427772" OR [Internal Compound ID]="205338663" OR [Internal Compound ID]="223082559") AND [Organism Name] !=(fulltext) "human") AND ([Relation Strength] = "Strong")
